# Prolonged Infection of Canine Distemper Virus in a Mixed-Breed Dog

**DOI:** 10.3390/vetsci8040061

**Published:** 2021-04-11

**Authors:** Zsófia Lanszki, Brigitta Zana, Safia Zeghbib, Ferenc Jakab, Nikoletta Szabó, Gábor Kemenesi

**Affiliations:** 1 National Laboratory of Virology, Szentágothai Research Centre, University of Pécs, H-7624 Pécs, Hungary; lanszkizsofi@gmail.com (Z.L.); brigitta.zana@gmail.com (B.Z.); zeghbib.safia@gmail.com (S.Z.); jakab.ferenc@pte.hu (F.J.); 2 Institute of Biology, Faculty of Sciences, University of Pécs, H-7624 Pécs, Hungary; 3 Corden International Hungary Ltd., 1117 Budapest, Hungary; vet@corden.hu

**Keywords:** *Paramyxoviridae*, *Morbillivirus*, canine distemper virus, haemagglutinin gene, arctic-like lineage, domestic dog, shelter, heartworm, *Dirofilaria immitis*

## Abstract

Canine distemper virus (CDV) is a major viral pathogen in domestic dogs, belonging to the *Paramyxoviridae* family, in the *Morbillivirus* genus. It is present worldwide, and a wide range of domestic animals and wild carnivores are at risk. In the absence of vaccination, dogs have a low chance of survival; however, if and when a dog survives, it can take an average of a few weeks to a few months to fully wipe out the virus. In the present study, we traced the course of infection of a 1-year-old mixed-breed male dog. The animal had an unusually long course of persistent CDV infection with a vector-borne heartworm (*Dirofilaria immitis*) co-infection. The dog excreted the CDV for 17 months with PCR positivity in urine samples collected from February 2019 through June 2020. The sequencing and phylogenetic analysis of the hemagglutinin gene revealed the CDV to be the member of the endemic Arctic-like genetic lineage. To the best of our knowledge, this report represents the longest documented canine infection of CDV. Notably, we highlight the necessity regarding CDV infectivity studies to better comprehend the transmission attributes of the virus.

## 1. Introduction

Canine distemper virus (CDV) is one of the most contagious viral agents among domestic dogs. It poses a significant conservation threat to a wide range of endangered animal populations around the world. It threatens a wide range of domestic and wild animals and can cross species barriers [1,2]. This virus is a significant veterinary health concern in areas in which the ratio of unvaccinated dogs is high and where the virus is also prevalent among wildlife. Young dogs are most commonly infected, but all ages are prone to infection and may quickly fall victim to the disease. The virus is primarily transmitted among dogs via various bodily fluids, such as respiratory droplets, saliva, urine and feces, including transmission with direct contact [3].

In consideration of the highly contagious nature of this virus, strict quarantine is required in cases of positivity until the effective clearance of CDV to avoid the spread of the virus to other animals [4]. If the infection reaches the central nervous system, it can lead to death [5]. In contrast, if a dog develops a strong immune response, the animal can completely recover from the infection [4,6]. Clinical signs characteristic of CDV in dogs may include gastrointestinal (vomiting, nausea and diarrhea), respiratory (nose, trachea and pneumonia) or neurological symptoms (mental dullness, lethargy, unresponsiveness, disorientation, blindness, imbalance and seizures) and fever. Among the CDV infections, more than 50% are likely subclinical, depending on the virulence of the virus strain, environmental conditions, host age and immune status [3,7]. Among dogs which survive the infection, the CDV is usually excreted for a few weeks; however, in some cases, the virus persists for up to 3–4 months [3,4,8]. The lengthy duration can weaken the immune system and in certain cases contribute to other superinfections or co-infections with other viruses, bacteria or cellular parasites [3,4,9,10].

Canine distemper virus is a single-stranded negative-sense RNA virus which belongs to the *Paramyxoviridae* family in the *Morbillivirus* genus. Along with the measles virus in humans, CDV is considered the most contagious virus in this family [11,12]. The CDV genome is approximately 15 kb and encodes six structural proteins: two glycoproteins, hemagglutinin (H) and fusion (F) proteins, one envelope-associated matrix (M) protein, two transcriptase-associated proteins phosphoprotein (P) and large polymerase (L) protein and one nucleocapsid (N) protein [6,13]. Several distinct genotypes are known and classified according to different hosts and geographical areas. This classification is based on nucleotide sequence analysis of the hemagglutinin (H) gene, which now indicates 17 lineages with characteristic phylogeographic distribution patterns [12]. The Arctic-like, Europe and European wildlife lineages have been reported throughout Hungary [14,15]. The hemagglutinin protein is a major fusogenicity determinant and plays a key role in the host-specific immunity against CDV [2,12,16,17,18,19].

Currently, approved CDV vaccines are based on attenuated virus strains. Most commercially available modified live CDV vaccines still belong to the America-1 lineage; therefore, many dogs immunized with this vaccine are prone to new CDV infections worldwide. Due to the high genetic diversity of circulating CDV strains, there might be significant antigenic differences which may lead to vaccine escape cases, as was hypothesized in multiple studies [5,12,17,19,20,21,22,23].

Here, we present the observation of 17 month CDV positivity in a mixed-breed dog in Hungary. We received only urine samples of the animal, which was previously described in other studies as the most suitable material for diagnostic purposes of CDV [24,25,26]. Additionally, we present the phylogenetic characterization of the observed virus strain. To our best knowledge, this is the most prolonged persistent CDV infection reported to date.

## 2. Materials and Methods

### 2.1. Case History

This report describes the case of a 1-year-old mixed-breed male dog (born in December 2017) afflicted with CDV infection in Hungary. The dog was kept in poor conditions and therefore was confiscated from its owner and transported to the Noah’s Ark Animal Shelter Foundation in Budapest. In all likelihood, the animal was infected with the virus prior to being transported to the shelter. Unfortunately, we do not have information regarding the vaccination history of the animal and whether it received the CDV vaccination or not.

On 27 January 2019, the dog was taken to the veterinary clinic with severe diarrhea and difficulty breathing. During the general health inspection of rescued animals, the *Dirofilaria immitis* infection was discovered with the WITNESS Canine Heartworm Antigen Test (Zoetis). On 1 February 2019, the animal’s symptoms improved, and the dog was returned to the shelter quarantine department. In the week following (8 February 2019), the animal was once again taken back to the veterinarian, this time with an intensive nasal discharge, cough and weight loss. The first CDV PCR test was performed with urine. Subsequently, the animal’s condition improved steadily and rapidly, requiring no further medical attention. Within a few days, the animal became entirely asymptomatic, and on 14 February 2019 was again placed in the quarantine department of the shelter until the animal was declared healthy, in August 2020, after the PCR confirmed the viral clearance from the animal’s urine.

### 2.2. PCR Reaction and Sequencing

The urine samples of the dog were collected multiple times, from February 2019 through August 2020. The total RNA was extracted using a Quick-RNA MiniPrep kit (Zymo Research, Irvine, CA, USA). In consideration of molecular detection regarding CDV, RNA samples were tested using real-time RT-PCR as formerly described [13]. Afterwards, to obtain a more representative genome segment for phylogenetic reconstruction, we applied additional PCR, targeting the hemagglutinin (H) gene of CDV with primer sets as previously published [27]. Due to multiple unsuccessful PCR amplification attempts, the primer pair (472f and 1172r) was replaced by newly designed primers (649f: 5’-CGCCTAGTAAGATCAAAGTG-3’ and 1216r: 5’-ACTTGATCCATAGGTGTTGC-3’). All PCRs were performed using the QIAGEN One-Step RT-PCR Kit (Qiagen, Germany) in full compliance with the manufacturer’s recommendations. For internal control (IC), we used the EIPC-K9 target, which is a common IC system in canine diagnostics [28]. For negative PCR control, we used nuclease-free water, whilst the positive control was a previously CDV-positive sample. The RT-PCR standard curve was generated by serial 10-fold dilutions of a corresponding CDV amplicon with a known copy number in a range of 1 × 10^10^ to 1 × 10^1^. These dilutions were measured in triplicate, and the measurement results were used to construct the standard curve, which was then used to determine the copy number from threshold cycle values (Ct) of the clinical samples. Final amplicons were sequenced using BigDye Terminator v1.1 Cycle Sequencing Kit in full accordance with the manufacturers’ protocol regarding ABI Prism 310 DNA Sequencer platform (Applied Biosystems, Foster City, CA, USA).

### 2.3. Phylogenetic Analysis

Prior to the phylogenetic reconstruction, cognate sequences were retrieved from the GenBank database and aligned with our sequence in the MUSCLE alignment webserver [29]. The final dataset comprised 59 either complete or partial H gene sequences; the final sequence length was 1824 nucleotides. Subsequently, the maximum likelihood phylogenetic tree was constructed under the Tamura 3-parameter with gamma-distributed rate heterogeneity (T92 + G) substitution model and 1000 bootstrap replicates using MEGA X software v.11 [30]. The resultant tree was then edited using iTOL (Interactive Tree of Life online platform (https://itol.embl.de/ (accessed on 1 March 2021)) [31].

## 3. Results

### 3.1. PCR Detection and Sequencing

The urine samples were PCR-positive in February, March, April, May, July, August, September, October and November 2019 and January, February and June 2020 and finally tested negative in August 2020. Viral genomic copy numbers are summarized in Table 1. The dog excreted the virus RNA for 17 months, which we successfully verified via PCR.

The full-length hemagglutinin (H) nucleotide sequence (2044 nt) were sequenced and submitted to GenBank database (accession number: MW248101). Based on the GenBank BLASTn search, the sequence depicted the highest nucleotide similarity (99.34%) with a representative sequence of the CDV Arctic-like lineage (KR002657) identified previously from a domestic dog which was transported from Hungary to Switzerland in 2013 [4]. It displayed 99.23% nucleotide identity with the KX943323 sequence that originated from a domestic dog in 2015 [32]. Similarly, 99.01% identity was observed with a sequence characterized from Apennine Wolves (*Canis lupus italicus*) in 2013 (KC966928) [33]. Likewise, it shared 98.52% and 98.83% identity regarding the nucleotide level with Italian sequences obtained from domestic dogs in 2005 (DQ226088) [20] and 2008 (HM443706) [34], respectively. It further represented a high nucleotide similarity 98.19% to an Arctic-like lineage formerly described in a domestic dog (DQ889184) from Hungary in 2005 [14].

### 3.2. Phylogenetic Analysis

**Figure 1:**
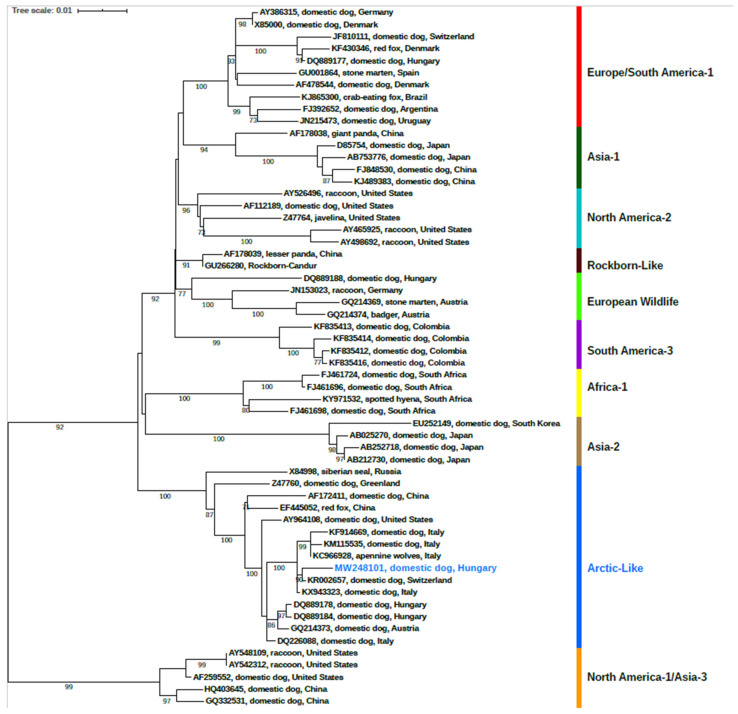
Phylogenetic tree based on the full-length hemagglutinin (H) nucleotide sequences. Phocine distemper virus (PDV) (GenBank accession number: AF479277) was used as an outgroup to root the phylogenetic trees. Bootstrap values lower than 70% are not shown. The sequence of interest is highlighted in bold and blue color.

Based on the phylogenetic analysis of all known genotypes (Figure 1.), the CDV strain explored in this study belongs to the Arctic-like genetic lineage of the virus. It is positioned in the genetic cluster of previously reported CDV sequences from Italy, Austria, Hungary and Switzerland. Considering that every CDV vaccines use the American lineage, we were able to exclude the possibility of vaccine-related positivity [35].

## 4. Discussion

This study presents clinical and the viral genetic aspects regarding a 17 month long persistent CDV infection in a sheltered dog which was symptomatic for a month with characteristic symptoms of acute CDV infection. This was followed by an asymptomatic phase with persistent CDV-positive PCR tests for a prolonged period (Table 1). The animal was quarantined at a veterinary clinic, and during this period we investigated the CDV RNA presence in the urine samples. Urine samples were previously described in other studies as the most suitable diagnostic material for CDV diagnostics [24,25,26] A clinical and molecular investigation from Switzerland was presented detailing how long dogs endure the viral RNA. Distinctly, the canine distemper virus infection was tested in a group of rescue dogs transported from Hungary to Switzerland, in which 11 of 13 dogs tested positive, and the virus was detected for a period up to four months before all dogs became PCR negative. The CDV isolates belonged to the Arctic-like lineage. The study highlighted the risks of unvaccinated dogs or dogs with incomplete vaccination [4]. According to several authors, the virus can be excreted up to 60 to 90 days following infection, although shorter shedding periods are more typical [3]. Viral shedding in feces may continue for up to 3–4 months; however, it usually resolves after 1–2 weeks [8]. There are few studies related to the rate of survival of infection. In an Australian study, 10 out of 13 dogs perished or were euthanized, and three out of 13 dogs recovered between 2006 and 2014. Admittedly, tjos represented a high (77%) mortality rate [7]. Moreover, In Italy, three out of 10 dogs recovered between 2015–2016, leading to a mortality rate of 70%. Additionally, the CDV isolates were associated to the Arctic-like lineage. Out of the 10 positive dogs, two were vaccinated, five were not vaccinated and three had an unknown vaccination status. The genetic heterogeneity of field and vaccine strains may lead to vaccinated canine infections, as was previously discussed [32]. However, we do not know the vaccine history of the animal in this study; thus, we present genetic data about the circulation of the CDV strain in Hungary [32].

There is a considerable lack of knowledge regarding the effect of other infections on CDV pathogenesis. CDV co-infection or superinfection cases with other pathogens have been previously reported, for instance, with viruses; canine adenovirus type 2 (CAV-2), canine influenza virus (CIV), canine parainfluenza virus (CPIV) [9,10] and bacteria—*Bordetella bronchiseptica*, *Mycoplasma cynos* [9,36]. There are further reports with vector-borne infections and co-infections with CDV, such as Babesia spp. and *Leishmania infantum* [4]. *D. immitis* is considered an endemic parasite with increasing relevance in Hungary. The first confirmed autochthonous canine heartworm case was detected in 2007 [37,38]. Nonetheless, we have limited information regarding co-infection with CDV and no data on the potential cross activity of non-specific immune response and the effect of parasitic infection in the course in reference to CDV infection, if any.

The Arctic-like lineage was initially detected among the susceptible population of the Arctic ecosystem in the 1980s [16,39,40,41]. This lineage was isolated from a Siberian seal (*Phoca sibirica*) in 1987–1988 from Lake Baikal in Russia [42]. Since then, the presence of Arctic-like lineage has been reported throughout many European countries including Italy, Hungary, Switzerland, Austria and other regions from North America, Iran and China [4,15,20,32,34,43,44,45,46]. This highlights the dominance and veterinary health relevance in certain geographic regions of the Artic-like lineage. The Arctic-like lineage is a widespread, relatively common strain across Europe among both domestic and wild animals, based on the available published literature. In Austria, from 2002 to 2007, 14 dogs, one badger (*Meles meles*) and one stone marten (*Martes foina*) were diagnosed with CDV. Three of the 14 dogs survived and were released to home care. Four different lineages were detected during the period of investigation, including one seven-year-old dog that was sample vaccinated for two and a half years with the Arctic lineage from 2003; it was euthanized [47]. In Southern Italy, nine CDV strains were sequenced between 2000 and 2004. Four CDV strains from dogs belonged to the European strains, two CDV strains from dogs belonged to the Arctic lineage (one was vaccinated, the other was not) and one CDV strain from a fox belonged to the European lineage. It was assumed that the uncontrolled trading of dogs likely introduced the Arctic-like strain into Italy from Eastern Europe or Northern Asia, and that strain steadily diffused and spread across the canine population [20]. At nearly the same time, 21 sequences belonging to the Arctic-like lineage were identified in north-eastern Italy, between 2000 and 2008, whereas five were related to the Europe 1 lineage from domestic dogs [34]. In another Italian study, diagnoses of 20 Apennine wolves (*Canis lupus*) with the CDV virus and their subsequent demise were reported in 2013. Only four of the 20 infected wolves underwent H gene sequencing and a subsequent phylogenetic classification to the Arctic lineage. This was the first report regarding CDV Arctic lineage epidemics among the wild population in Europe [33]. Following that, two badgers sampled from a rural area in Italy in 2015 were reported to be CDV-positive with PCR. The CDV isolates were lethal in badgers and belonged to the Arctic-lineage based on the H protein. Since the badgers came from the same area as the Apennine wolves, these results likely suggest that this lineage is becoming endemic in the wildlife population of the Abruzzo region, Italy [48]. In particular, between 2015 and 2016, the Arctic-like lineage was still detectable throughout Italy. The infection data from recent years supports the theory that the introduction of the Arctic-like lineage was carried by dogs imported from Eastern European countries and suggests the possibility of a potential common origin [32].

In a previous Hungarian study, the authors showed the diversity of the Hungarian CDV strains. According to the results, several different CDV genotypes were present at the same time in the country. The phylogenetic analysis was performed with the full segment of the H gene nucleotide sequence. Thirteen samples from dogs were selected for sequence analysis between 2004–2006. Nine sequences belonged to the group of Arctic-like strains, one group belonged to the European isolates cluster and three belonged to the European wildlife group; all sequences were from dogs. The nine dogs had unknown vaccination histories. Additionally, CDV infection was also detected in other carnivores, such as fox (*Vulpes vulpes*), raccoon (*Procyon lotor*) and ferret (*Mustela putorius furo*); however, these species were not involved in any genetic analysis [14,15]. Since this strain was detected in Hungary as early as 2004, it may since have become endemic. Large-scale epidemiological surveys are required to obtain a general picture of the prevalence, geographic distribution and seasonality of the Arctic-like lineage within our region.

## 5. Conclusions

Canine distemper is a serious viral disease that affects many species around the world. Among dogs, it is associated with high rates of morbidity and mortality, although not necessarily leading to a fatal outcome. Surveillance and strict quarantine measures are crucial in preventing the spread of CDV infection. Many lineages have been present in recent decades, including the Arctic-like lineage. Sequencing and subsequent phylogenetic analysis have demonstrated that, in the case related in this work, the CDV belonged to the Arctic-like lineage. This lineage is known to circulate throughout Southern and Central Europe amongst domestic dogs and wildlife. Our study highlights the need for an effective surveillance system for CDV to monitor the prevalence and the distribution of the virus.

To the best of our knowledge, this is the first report referencing a prolonged Canine distemper virus infection for 17 months in association with the Arctic-like lineage and the first reported co-infection with a *Dirofilaria immitis* parasite. Additional studies are necessary to fully understand the Arctic-like CDV strains and their prevalence among dog populations and evaluate any potential CDV spillover. It is also required to extend targeted surveillance in future studies of the virus towards wildlife specimens, considering the examples of spillover events. Complex studies in the future following the One Health concept may reveal the background mechanisms of cross-species transmission events and the risk of infection from wildlife. A major limitation of our study is the lack of infectivity studies; however, merely considering the PCR results, it is a notable case of CDV infection.

## Figures and Tables

**Table 1:** Detection of canine distemper virus (CDV) RNA by real-time RT-PCR with corresponding viral genomic copy numbers.

	2019									2020		
	February	March	April	May	July	August	September	October	November	January	February	June
Ct value	18.48	22.66	20.37	23.9	32.66	39.75	38.3	39.3	40	39.39	40	41
cRNA (*n* copies/μL)	2,510,955	207,819	813,841	99,235	536	<100	<100	<100	<100	<100	<100	<100

## Data Availability

Data is contained within the article.
